# Supplementary material for: Individual-, household- and community-level determinants of infant mortality in Ethiopia
Source: PLoS One. 2021 Mar 12;16(3):e0248501. doi: 10.1371/journal.pone.0248501 (PMC7954351; doi:10.1371/journal.pone.0248501)
Supplement: Attachment — Submitted filename: Reviiew report for paper.docx [file pone.0248501.s001.docx]

**Overall Assessment:** The manuscript is well conceived and executed and makes an important contribution to understanding the underlying causes of infant mortality in Ethiopia.

**Introduction:**

The authors state that the Ethiopian Demographic and Health Survey (EDHS) 2016 has been analyzed for infant mortality before by other researchers. However, they make the case that all earlier studies have examined individual level factors only and did not pay sufficient attention to community level factors. The authors argue, convincingly why other studies failed to take into account shared community level characteristics, which make a significant contribution to high infant mortality rates; they rightly say that these need to be taken into account when considering infant mortality rates, given the objectives attaining SDG 3 goals of reducing infant mortality rates. The literature review section on this aspect is thorough and point to numerous community level factors that should be taken into account while discussing infant mortality in Ethiopia.

**Materials and Methods**

The method section is straight forward and clearly written, describing the study variables and the community characteristics. While Table 1 appears to be very cumbersome, all the information Is there. The table captures information pertaining to parameters for analysis, including, antenatal care, place of birth, number of adverse pregnancy events, skilled delivery (referring to a doctor, nurse or a midwife). Postnatal checkups, tetanus shots, age of partner, household wealth index, distance from health facility. This table also included the community level factors such as region of residence, quality of water available, sanitation facilities, multidimensional poverty indices. These items were recoded for analysis, recoding of these items.

**Results and Data Analysis:**

The results (tables 2-7) describe the general characteristics of the study population at the individual and community level. The data analyses consist of descriptive analysis section and a multi-stage multivariate analysis. It requires fairly sophisticated analytical skills. Some of the results are consistent with findings by other investigators. However, they have managed to tease out information that is important.

Suggestions to improve readability.

1. Line 85: - which could be introduces a serious bias - is grammatically wrong. It should read as follows: **which could introduce a serious bias**;
2. Line 111: the last sentence is a bit confusing need to make sure that the reference is quoted correctly;
3. Line 158: replace -that share with **who share**;
4. Table 1, page 6: there needs to be an explanation of what **century day code** means in the context of this study;
5. Table 1 Page 7 reported birth size: It cannot be three fourths (3/4ths) of the births but two thirds (2/3) of the births;
6. Table 1, page 7, reported birth size: **delete** **were** from the sentence;
7. Table 1, page 7 reported birth size delete **were** and write **gave birth**;
8. Table 1, total live births replace **much** with **many**;
9. Table 1 page 7: need to pay attention to tense; since past tense was used earlier, I suggest using **was** instead of **is**;
10. Table 1: page 8 age of **mother** instead of **mother'**s;
11. Table 1, Marital status page 8 **marital status** and not **martial** status;
12. Table 1, page 8: marital status - it seems that the categories did not follow what was indicated in the text. Suggest rewriting this section to avoid confusion;
13. Table 1, number of adverse pregnancy events, page 8: **replace child** with **infant** in this phrase;
14. Table 1, page 9: should this be the last pregnancy? Please clarify
15. Table 1, place of birth, page 9: this phrase needs to be edited: place where mothers **gave** not **give**. the word **origination** needs to be replaced;
16. Table 1, number of adverse pregnancy events, page 10: replace **child** with **infant** in this phrase;
17. table 1, skilled delivery, page 10: **insert was** in this sentence:
18. Table 1, partner age, page 11: were husbands always older than the wife?
19. Table 1, House-hold wealth, page 11: index the correct spelling; this should be **principal** and not **principle**;
20. Table 1, distance from health facility, page 11: **was** instead of **were**;
21. Table 1, distance from health facility, page 11: **rating** not **rate**;
22. Table 1, distance from the health facility, page 12: **add after** self report
23. Table 1, distance from health facility, page 12: **options** not **option**
24. Table 1, distance from the health facility, page 12: delete it from the sentence to read as is.
25. Table 1, region of residence, Page 12: replace **who** with **that**
26. Line 204: **had** instead of **have.**
27. Line 210: should be - **at an age.**
28. Line 214: majority **of** the head of households were males
29. Line 284: delete **a**
30. Line 287: The authors have only considered female genital mutilation as a contributing factor. They have not considered the impact of male child preference and neglect of female infants. In India for example, female infant mortality or sex ratio favors male infants. Suggest paper by Prabhat Jha <https://arthaimpact.com/wp-content/uploads/2019/09/bc574888-cd46-4f7b-afc2-8d024829057b_35.pdf>
31. Line 298: Multiple births have a huge impact on the on the health of the pregnant woman during the perinatal period, including deliveries. I wonder if the authors attempted to correlate C-sections with multiple pregnancies.
32. Line 302: **insert a comma** after the word **increased.**
33. Line 303: remove **was** from this sentence
34. Line 305: rephrase the ....lower among women who attended four and more antenatal care visits
35. Line 308: **change sentence** compared to women (mothers) who had no such previous loss
36. Line 309: I suggest deleting the sentence Studies ...on the survival of the next infant OR add the references to this statement
37. Line 311: I suggest changing the word **survivorship** to **survival**. The meaning of biologically and environmentally is not clear in this context. I suggest removing this altogether or provide more explanation as to why there is an environmental concern.
38. Line 314: **insert of** before breastfeeding
39. Line 316: I am not familiar with maternal depletion syndrome please include a description.
40. Line 323: do you mean 4.1% instead of 41.%?
41. Line 324 and 325: This sentence needs to be fixed, it is not grammatically correct
42. Line 326: I suggest changing the sentence to - but much higher than- instead of and also much higher compared to other African countries.
43. Line 327-329: these data seem a bit random since not all regions are compared with respect to family planning, immunization coverage and access to hospitals between Tigray, Somali and Afar regions. Could comparative statistics not be located for all the parameters??
44. Line 330: suggest changing the tense to past tense since that is the format used.
45. Line 336-337: this argument seems logical. However, it seems that there are people who belong to the rich category in this group. Was a separate analysis done for the group considered to be rich in infant mortality group?
46. Line 344: the most important reason why infant mortality is high in the mountainous region is the distance from a health post where any assistance can be given to gravid women. Sometimes women have to walk or be carried physically over very difficult terrain to reach the health post. If there is obstructed labor or needing C-section, neither the woman nor the infant survives the journey. Some acknowledgment of this reality should be included in this text.
47. Lines 350-351 This sentence seems to contradict the main argument made in the paper that both community level and individual level factors affect infant mortality. I suggest modifying the statement somewhat.
48. Line 362-365: This could be a stronger statement than the one presented here. Clearly, as suggested in the paper, community level factors include health infrastructure with availability of health facilities and even antenatal services. These come well within the purview of the SDG goals and deserve a stronger recommendation as a conclusion of the analysis.
