# Supplementary material for: Individual-, household- and community-level determinants of infant mortality in Ethiopia
Source: PLoS One. 2021 Mar 12;16(3):e0248501. doi: 10.1371/journal.pone.0248501 (PMC7954351; doi:10.1371/journal.pone.0248501)
Supplement: Attachment — Submitted filename: Point by point response.doc [file pone.0248501.s002.doc]

Editor in Chief, PLOS One. Feb /2021

Article type: original article

**Subject:** A list of modifications and a response to the reviewers – revised manuscript **PONE-D-20-29562**

Dear Editor,

We appreciate the interest that the editors and reviewers have taken in our manuscript and the constructive criticism they have given. We have addressed the concerns of the reviewers and editors especially variable selection and model building. The changes concerning English are marked tracked in the manuscript. We have included a point-by-point response to the reviewers in addition to making the changes described above in the manuscript. Of course, because of the changes taking place, the numbering of lines both in the original and revised manuscripts was altered. The Author confrmied that the production will need reference to link the reader to the tables 2, 3, 4 and 5 in the text, if accepted. Finally, the authors received no specific funding for this work.

**Point by point response to reviewer’s comments**

|  | Reviewer’s comments | Author’s response |
| --- | --- | --- |
| 1 | a) please, explain in more detailed the exclusion of some community level factors, more detailed on the estimation of ICC and PCV for the logistic models. I would like to see more information on the presentation of each model. It is my understanding that the modeling strategy should first estimate a model that allows to decompose the variance between the levels of analysis was presented. It does not include explanatory variables, and the error terms act only on the dependent variable, allowing the partition of the total variability found in the data according to the levels of analysis. Based on the null model, more models could be developed, as there is great heterogeneity in the dependent variable. Was that the approach followed? | Thank you very much for these critical questions. We have added the following paragraph: **See line 172-174**  Backward stepwise multilevel logistic regression analysis was performed to select individual-, household- and community-level variables to each model and those variables with *p-value* > 0 .25 were removed.  Thank you very much for the critical observation regarding the null model. During model building in this analysis, the null model or model I does not have any contribution to the build the rest of models. In this analysis we started model building with a saturated model i.e. entering all possible variables and removing using a backward stepwise selection. Regarding ICC, it is the measure of variance (random effects), which is the measure of residual errors at individual level and community levels. In this study there was a variation in the odds of infant mortality across communities [1]. |
| 2 | Line 85: - which could be introduces a serious bias - is grammatically wrong. It should read as follows: **which could introduce a serious bias**; | We have made the suggested correction **(See line 88).** |
| 3 | Line 111: the last sentence is a bit confusing need to make sure that the reference is quoted correctly; | **We have made the following correction (See line 105-107).**  In a nationally representative cross-sectional study of 28,647 live births in Nigeria also showed that 16.7% of the variance in the risks of infant mortality across community level characteristics [2]. |
| 4 | Line 158: replace -that share with **who share**; | **We have made the appropriate correction and revision (See line 150- 153).**  These are the characteristics of a community or cluster. A community comprises of people living in a particular area or in a common location. In the 2016 Ethiopian Demographic and health survey programmes, the primary sampling units (PSU) are considered as proxies for communities or clusters [3]. |
| 5 | Table 1, page 6: there needs to be an explanation of what century day code means in the context of this study; | **We have added the following explanation to table 1, page 7).**  The century day code is analogous to the century month code and gives the number of days since the beginning of 1900. A century month code (CMC) is the number of the month since the start of the century. For example, January 1900 is CMC 1, January 1901 is CMC 13, and January 1980 is CMC 961[4]. |
| 6 | Table 1 Page 7 reported birth size: It cannot be three fourths (3/4ths) of the births but two thirds (2/3) of the births; | **We made correction, two third to three-fourth (See table 1 Page 7).**  Measuring the actual weight of the newborn child was very challenging because only one-fourth of Ethiopian mothers gave birth at health facilities; the remaining three-fourth of Ethiopian mothers gave birth at home |
| 7 | Table 1, page 7, reported birth size: **delete** **were** from the sentence; | We have made the appropriate correction **(see table 1, page 7).** |
| 8 | Table 1, page 7 reported birth size delete **were** and write **gave birth**; | We have made the appropriate correction **(see table 1, page 7).** |
| 9 | Table 1, total live births replace **much** with **many**; | We have made the appropriate correction **(see table 1, page 8).** |
| 10 | Table 1 page 7: need to pay attention to tense; since past tense was used earlier, I suggest using **was** instead of **is**; | **We have mad correction** **(see table 1, page 8).** |
| 11 | Table 1: page 8 age of **mother** instead of **mother'**s; | We have made the appropriate correction **(see table 1, page 8).** |
| 12 | Table 1, Marital status page 8 **marital status** and not **martial** status; | We have made the appropriate correction **(see table 1, Marital status page 8).** |
| 13 | Table 1, page 8: marital status - it seems that the categories did not follow what was indicated in the text. Suggest rewriting this section to avoid confusion; | We have added the definition of each categories**. (see table 1, Marital status page 8).**  The original question asked for the marital status of the respondents, and the response options were ‘never married’, ‘married’, ‘living together’, ‘separated’ and ‘widowed’[5]. In this study, ‘never married persons’ are persons who never got married in concordance with valid regulations. “Married persons’ are those who got married before a [competent body](http://ec.europa.eu/eurostat/en/web/products-manuals-and-guidelines/-/KS-GQ-15-002) in concordance with valid regulations. “Widowed persons” are persons whose marriage ceased to exist by death of one of spouses or by declaring a missing spouse dead respectfully. “Divorced persons” are those whose marriage was terminated. “Separated persons” had previously lived with a partner but were not currently living with a partner. ‘living together persons’ are those who living together but have not valid regulations, [6, 7]. |
| 14 | Table 1, number of adverse pregnancy events, page 8: **replace child** with **infant** in this phrase; | We have made the appropriate correction **(see table 1, page 9).** |
| 15 | Table 1, page 9: should this be the last pregnancy? Please clarify | We have made the appropriate correction **(see table 1, page 9).**  We changed the phrase this pregnancy to the last pregnancy. |
| 16 | Table 1, place of birth, page 9: this phrase needs to be edited: place where mothers **gave** not **give**. the word **origination** needs to be replaced; | We have made the appropriate correction **(see table 1, page 9).** |
| 17 | Table 1, number of adverse pregnancy events, page 10: replace **child** with **infant** in this phrase; | We have made the appropriate correction **(see table 1, page 9).** |
| 18 | table 1, skilled delivery, page 10: **insert was** in this sentence: | We have made the appropriate correction **(see table 1, page 10).** |
| 19 | Table 1, partner age, page 11: were husbands always older than the wife? | Thank you very much for this important question. We have made the following revision. (**See table 1, partner age, page 10)**  The participants’ and their partners’ ages were used to calculate the difference in age. The age difference among partners was categorized as: ‘husband older by less or equal to five years’, ‘husband older by five years or more’, Wife older by less or equal to five and wife older by five years [6, 7]. However, in this study all husband were older than wife |
| 20 | Table 1, House-hold wealth, page 11: index the correct spelling; this should be **principal** and not **principle**; | We have made the appropriate correction **(see table 1, page 10).** |
| 21 | Table 1, distance from health facility, page 11: **was** instead of **were**; | We have made the appropriate correction **(see table 1, page 10).** |
| 22 | Table 1, distance from health facility, page 11: **rating** not **rate**; | We have made the appropriate correction **(see table 1, page 10).** |
| 23 | Table 1, distance from the health facility, page 12: **add after** self report | We have made the appropriate correction **(see table 1, page 11).** |
| 24 | Table 1, distance from health facility, page 12: **options** not **option** | We have made the appropriate correction **(see table 1, page 11).** |
| 25 | Table 1, distance from the health facility, page 12: delete it from the sentence to read as is. | We have made the appropriate correction **(see table 1, page 11**). |
| 26 | Table 1, region of residence, Page 12: replace **who** with **that** | We have made the appropriate correction **(see table 1, page 11).** |
| 27 | Line 204: **had** instead of **have.** | We have made the appropriate correction **(see line 188).** |
| 28 | Line 210: should be - **at an age.** | We have made the appropriate correction **(see line 194).** |
| 29 | Line 214: majority **of** the head of households were males | We have made the appropriate correction **(see line 197).** |
| 30 | Line 284: delete **a** | We have made the appropriate correction (**see line 266**).` |
| 31 | Line 287: The authors have only considered female genital mutilation as a contributing factor. They have not considered the impact of male child preference and neglect of female infants. In India for example, female infant mortality or sex ratio favors male infants. Suggest paper by Prabhat Jha <https://arthaimpact.com/wp-content/uploads/2019/09/bc574888-cd46-4f7b-afc2-8d024829057b_35.pdf> | Thank you very much for this very important critique and the material  We have added the following paragraph:  Another reason for the high rate of infant mortality in females compared with males may be due to sex preference. For families in Asia and Africa, a preference for sons is common [8]. For some families, sons are preferred as they have a higher wage-earning capacity (especially in agrarian economies) and can take care of parents in later life [9]. For example, in a study from a national survey in India on child gender and parental investment, researchers found that boys received an average of 10% more time and care from their parents than girls did [10]. Another study on Gender and cross-cultural dynamics in Ethiopia also presented that only 20.7% of the study percipients preferred female children [11]. **See** **line 273-281** |
| 32 | Line 298: Multiple births have a huge impact on the on the health of the pregnant woman during the perinatal period, including deliveries. I wonder if the authors attempted to correlate C-sections with multiple pregnancies. | Thank you for this suggestion. In this study C-sections were highly correlated with multiple pregnancies at p-value < 0.001 and for this reason we did not include both variables in the model. We chose to include the multiple birth variable in the model based on the bivariate analysis. |
| 33 | Line 302: **insert a comma** after the word **increased.** | We have made the appropriate correction **(see line 292).** |
| 34 | Line 303: remove **was** from this sentence | We have made the appropriate correction **(see line 293).** |
| 35 | Line 305: rephrase the ....lower among women who attended four and more antenatal care visits | We have rephrased as the following: For example, a systematic review in Ethiopia indicated that the risk of early infant death was lower among women who had four or more ANC visits compared to those who had less than four visits [12]. **See line 296-298)** |
| 36 | Line 308: **change sentence** compared to women (mothers) who had no such previous loss | We have changed the sentence to read: The findings from this study showed that women (mothers) who had experienced a previous infant loss were twice as likely to experience a subsequent infant death compared to mothers who had no such previous loss. **(see line 296-298**) |
| 37 | Line 309: I suggest deleting the sentence Studies ...on the survival of the next infant OR add the references to this statement | We have added a reference to this statement **(see line 299).** |
| 38 | Line 311: I suggest changing the word **survivorship** to **survival**. The meaning of biologically and environmentally is not clear in this context. I suggest removing this altogether or provide more explanation as to why there is an environmental concern. | We have made the appropriate correction **(see line 300).** |
| 39 | Line 314: **insert of** before breastfeeding | We have mad correction **(see line 303).** |
| 40 | Line 316: I am not familiar with maternal depletion syndrome please include a description. | **We have added the following paragraph (see line 306-310).** Although the term “maternal depletion syndrome (MDS)” is used to describe the poor health status of mothers, however whether such a syndrome actually exists remains unclear [13]. The syndrome was commonly assigned to the nutritional stress induced by successive pregnancies, and pregnancies that were close together [13]. |
| 41 | Line 323: do you mean 4.1% instead of 41.%? | Thanks, we have mad correction it is 4.1% **(see line 317).** |
| 42 | Line 324 and 325: This sentence needs to be fixed, it is not grammatically correct | We have made the following correction **(See line 316-317).**  Universal Health Coverage (UHC) was lowest in Somali and Afar regions at 3.7% and 4.1%, respectively [14]. |
| 43 | Line 326: I suggest changing the sentence to - but much higher than- instead of and also much higher compared to other African countries. | We have made the appropriate correction **(see line 323).** |
| 44 | Line 327-329: these data seem a bit random since not all regions are compared with respect to family planning, immunization coverage and access to hospitals between Tigray, Somali and Afar regions. Could comparative statistics not be located for all the parameters?? | We used the findings from the descriptive national health service coverage report, and estimates were not available for all parameters. |
| 45 | Line 330: suggest changing the tense to past tense since that is the format used. | We have made the appropriate correction **(see line 323).** |
| 46 | Line 336-337: this argument seems logical. However, it seems that there are people who belong to the rich category in this group. Was a separate analysis done for the group considered to be rich in infant mortality group? | We used a wealth index to measure the household income and categorized as poor, middle (average) and rich. Unfortunately the income status of the household is not significantly associated with infant mortality in this study. |
| 47 | Line 344: the most important reason why infant mortality is high in the mountainous region is the distance from a health post where any assistance can be given to gravid women. Sometimes women have to walk or be carried physically over very difficult terrain to reach the health post. If there is obstructed labor or needing C-section, neither the woman nor the infant survives the journey. Some acknowledgment of this reality should be included in this text. | Thank you very for this very important comment.  **We have added the following paragraph (See line 340-345).**  In addition, living in mountain areas makes access to health services difficult. For example, a study conducted in rural areas of Ethiopia found that people living in remote areas are at high risk of child mortality [15]. Children who lived 1.5 hours or more from a health facility were at a two-fold higher risk of death compared to those who lived within 1.5 hours from a facility [15]. The reason may be that people living in mountainous and remote areas may spend many hours travelling by foot to access maternal and infant health services. |
| 48 | Lines 350-351 This sentence seems to contradict the main argument made in the paper that both community level and individual level factors affect infant mortality. I suggest modifying the statement somewhat. | We have mad correction **(see line 346-351)** |
| 49 | Line 362-365: This could be a stronger statement than the one presented here. Clearly, as suggested in the paper, community level factors include health infrastructure with availability of health facilities and even antenatal services. These come well within the purview of the SDG goals and deserve a stronger recommendation as a conclusion of the analysis. | Thank you very much.  **We have added the following sentence (see line 390-393).**  Finally, we recommended that policy makers and governments focus on community level factors in addition to individual and household level factors to achieve the SDG goals and targets by the end of 2030. |

1.	Merlo, J., et al., *A brief conceptual tutorial of multilevel analysis in social epidemiology: linking the statistical concept of clustering to the idea of contextual phenomenon.* Journal of Epidemiology & Community Health, 2005. **59**(6): p. 443-449.

2.	Adedini, S.A., et al., *Regional variations in infant and child mortality in Nigeria: a multilevel analysis.* Journal of biosocial science, 2015. **47**(2): p. 165-187.

3.	CSA, I., *Ethiopia demographic and health survey 2016.* Addis Ababa, Ethiopia and Calverton, Maryland, USA: Central Statistical Agency and ICF International, 2017. **551**.

4.	Team, S.M.M., *The DHS Program User Forum.*

5.	Schoenborn, C.A., *Marital status and health, United States 1999-2002*. 2004: US Department of Health and Human Services, Centers for Disease Control and ….

6.	MEASURE, D., *ICF International. Standard recode manual for DHS 6 (version 1.0); description of the Demographic and Health Surveys individual recode data file. Measure DHS, Calverton, Maryland. Calverton, Maryland: MEASURE DHS/ICF International; 2013*.

7.	CSA, I., *Ethiopia demographic and health survey 2016*. 2017: Addis Ababa, Ethiopia and Calverton, Maryland, USA: Central Statistical Agency and ICF International. p. 24.

8.	Das Gupta, M., et al., *Why is son preference so persistent in East and South Asia? A cross-country study of China, India and the Republic of Korea.* The Journal of Development Studies, 2003. **40**(2): p. 153-187.

9.	Hesketh, T. and Z.W. Xing, *Abnormal sex ratios in human populations: causes and consequences.* Proceedings of the National Academy of Sciences, 2006. **103**(36): p. 13271-13275.

10.	Barcellos, S.H., L.S. Carvalho, and A. Lleras-Muney, *Child gender and parental investments in India: Are boys and girls treated differently?* American Economic Journal: Applied Economics, 2014. **6**(1): p. 157-89.

11.	Kifetew, K., *Gender and cross cultural dynamics in Ethiopia.* Agenda, 2006. **20**(68): p. 122-127.

12.	Wondemagegn, A.T., et al., *The effect of antenatal care follow-up on neonatal health outcomes: a systematic review and meta-analysis.* Public health reviews, 2018. **39**(1): p. 33.

13.	Winikoff, B. and M.A. Castle, *The maternal depletion syndrome: clinical diagnosis of eco-demographic condition?* The maternal depletion syndrome: clinical diagnosis of eco-demographic condition?, 1987.

14.	Eregata, G.T., et al., *Measuring progress towards universal health coverage: national and subnational analysis in Ethiopia.* BMJ Global Health, 2019. **4**(6).

15.	Okwaraji, Y.B., et al., *Effect of geographical access to health facilities on child mortality in rural Ethiopia: a community based cross sectional study.* Plos one, 2012. **7**(3): p. e33564.
